# Pulling in new directions: Myosin 2, Piezo, and metabolism

**DOI:** 10.12688/f1000research.18856.1

**Published:** 2019-08-22

**Authors:** Melissa A. Quintanilla, John A. Hammer, Jordan R. Beach

**Affiliations:** 1 Cell and Molecular Physiology, Loyola University Chicago, Stritch School of Medicine, Center for Translational Research and Education, Maywood, IL, USA; 2 Cell and Developmental Biology Center, National Heart Lung and Blood Institute, National Institutes of Health, Bethesda, MD, USA

**Keywords:** myosin, piezo, metabolism

## Abstract

Myosin 2 plays a central role in numerous, fundamental, actin-based biological processes, including cell migration, cell division, and the adhesion of cells to substrates and other cells. Here, we highlight recent studies in which the forces created by actomyosin 2 have been shown to also impact tension-sensitive ion channels and cell metabolism.

## Introduction

All cells sense their surrounding environment. A critical component of environmental sensing is sensing the mechanical environment, wherein cells convert mechanical information into biochemical information in a process known as mechanotransduction. Mechanotransduction is especially important for processes like differentiation, morphogenesis, and disease progression. A central mediator of mechanotransduction is the actomyosin cytoskeleton because it represents the principal contractile element of eukaryotic cells. Through its interaction with the actin cytoskeleton, non-muscle myosin 2 (hereafter referred to simply as “myosin 2”) serves as the dominant generator of contractile forces in all non-muscle cells and is intimately involved in numerous mechanotransduction pathways. (Please see recent reviews highlighting the general properties of myosin 2 ^1– 4^, myosin 2 assembly dynamics ^5, 6^, and roles for myosin 2 in mechanotransduction ^7, 8^.) In this short review, we focus on two emerging areas regarding the relationships between myosin 2, environmental sensing, and mechanotransduction: the connection between myosin 2–generated forces and the tension-sensing membrane channel Piezo1 and the connection between myosin 2–generated forces and cell metabolism.

## Myosin 2 and Piezo1

An important breakthrough in 2010 was the discovery of Piezo1 and Piezo2 ^9, 10^, the pore-forming subunits of the long-sought ion channels in the plasma membrane which open upon application of mechanical forces to cells, such as deformation or shear stress. In response to these external forces, Piezo channels open for several milliseconds to allow the entry of cations like calcium into the cytoplasm. Building on previous studies ^11– 13^, recent medium-resolution cryogenic electron microscopy (cryo-EM) images of Piezo1 reveal a beautiful trimeric structure composed of three curved blades that surround a central pore, which is topped by a cap-like domain ^14^. Even more interestingly, Piezo1 in its closed state appears to bend the surrounding plasma membrane inward, creating a sort of dimple in the membrane. Current thinking is that increased tension within the plasma membrane caused by mechanical deformation of the cell causes Piezo1 to assume a more planar shape, resulting in transient opening of its channel. Piezo1 is therefore a sensor of in-plane membrane tension that responds to increased tension by allowing the transient entry of calcium and other cations into the cytoplasm to promote myriad physiological processes.

Most studies to date have focused on the role of Piezo in response to external forces. These studies have revealed several fascinating examples where the response of Piezo to force influences cell behavior. For example, Piezo1 has been implicated in the maintenance of cell density in epithelial layers ^15, 16^. In undercrowded layers, Piezo1-dependent calcium fluxes in the stretched epithelium promote cell division to restore normal cell density, whereas in overcrowded layers, Piezo1, together with some additional signals, promotes cell extrusion/expulsion to restore normal cell density. Piezo has also been implicated in the control of axonal regeneration ^17^ and stem cell fate decisions ^18^. For example, the stretching or confining of *Drosophila* gut tissue controls via Piezo-mediated calcium transients the differentiation of progenitor cells into enteroendocrine cells ^18^. Along similar lines, Pathak *et al*. showed that Piezo1-mediated calcium fluxes in neural stem cells depend on substrate stiffness and influence the matrix stiffness–dependent decision made by these cells to differentiate into either neurons or astrocytes ^19^. Interestingly, these authors also showed that the Piezo1-mediated calcium transients seen on certain substrates require myosin 2–based contractility. This finding is consistent with the major role played by actomyosin 2–dependent force in sensing substrate stiffness and generating traction force though focal adhesions ^20^ and it argues that Piezo1 can be activated by cell-generated forces in addition to external forces.

A recent study from the Pathak lab provided additional evidence that myosin 2–based traction forces promote the opening of Piezo1; and the study showed that Piezo1 opening occurs only in the immediate vicinity of the traction site ^21^. Using fibroblasts isolated from a mouse in which Piezo1 had been tagged with a red fluorescent protein, the authors first showed that Piezo1 was uniformly distributed in the plasma membrane. This observation argues that spatially restricted Piezo1 activity is not a result of spatially restricted localization. (Of note, other studies using immunofluorescence techniques have observed conflicting data, in which Piezo1 ^22^ and Piezo2 ^23^ were enriched at focal adhesions.) After developing an imaging routine to visualize transient calcium “flickers” arising from Piezo1 channels in the ventral membrane, Ellefsen *et al*. co-imaged these calcium flickers and sites of traction force by using a Förster resonance energy transfer (FRET)-based traction sensor ^21^. This effort yielded two important results. First, Piezo1 channel opening is enhanced significantly by traction force. Second, this enhancement occurs only in the immediate vicinity of traction force sites. Inhibition of myosin 2 contractility using an inhibitor of myosin light chain kinase, a major activator of myosin 2, largely eliminated both traction force and the focal, Piezo1-dependent calcium flickers. Together, these observations argue that tension generated in the plasma membrane in the immediate vicinity of traction force sites (that is, focal adhesions subjected to actomyosin 2–dependent strain) opens adjacent Piezo1 channels to allow spatially restricted calcium entry.

Relevant to the new findings of Ellefsen *et al*., Shi *et al*. recently demonstrated that membrane tension arising from a focused deformation of the plasma membrane does not propagate across the entire cell surface as generally assumed based on previous studies using planar lipid bilayers ^24^. Instead, the tension is confined to the immediate vicinity of the deformation, mostly likely by transmembrane proteins and cytoskeletal/membrane linkages that impede membrane flow ^24^. This seminal observation argues that local increases in membrane tension lead to localized mechano-signaling, a conclusion completely in line with the new results from Ellefsen *et al*. More generally, the new results of Ellefsen *et al*. indicate that Piezo1 can be activated by internal, cell-generated forces as well as by applied external forces and that myosin 2 creates at least one such cell-generated force in the form of traction force. Given that myosin 2 represents the major contractile machine in most cell types, it seems likely that it drives local membrane tension, and consequently local Piezo1 activity, in many other biological contexts. Most importantly from a physiological standpoint, the resulting local calcium transients could regulate a wide variety of biological events, such as enzyme activation, cytoskeletal remodeling, and regulated vesicle secretion. Shi *et al*. showed that, consistent with this final point, vesicle secretion is in some instances enhanced proximal to sites of membrane tension development ^24^. Moreover, Piezo-mediated calcium transients caused by external pressure activate myosin 2 in *Dictyostelium* ^25^, creating a potentially robust feedforward loop.

## Myosin 2 and cell metabolism

Another area of growing interest is the interaction between cell mechanics and cell metabolism. Although the necessity for this interaction is obvious in muscle tissue, where actomyosin contractility is a major consumer of energy, it also appears to be important in non-muscle cells. An obvious mechanism to link cell mechanics and metabolism is through mechanosensitive transcription factors like YAP/TAZ and SRF. This connection was recently reviewed in depth for both normal and diseased tissues ^26^. Interestingly, recent studies suggest that cell mechanics can also have direct, non-transcriptional influences on cell metabolism. An oversimplified model of cell metabolism would employ a sensor of ATP levels, a mechanism to increase substrate utilization and ATP production by mitochondria when ATP levels are low, and a mechanism to slow ATP production and store substrates when ATP levels are high. Here, we highlight recent studies that suggest direct roles for actomyosin contractility in regulating all three components of this simplified model (that is, ATP sensing, mitochondrial homeostasis, and lipid storage), and we briefly review the intersection between mechanics and metabolism during cancer cell metastasis.

### ATP sensing

If mechanics and ATP sensing are mechanistically linked, then sensing could occur at sites where external forces are transduced across the cell membrane, such as at cadherin-based cell–cell contacts, integrin-based adhesions, and membrane protrusions ^27^. A central sensor of energy status is AMP kinase (AMPK), named for its activation upon binding AMP. When ATP is being used, the AMP that is simultaneously generated activates AMPK to stimulate energy production and downregulate unnecessary energy consumption or storage. A recent study by Bays *et al*. investigating the role of AMPK in epithelial cell mechanotransduction ^28^ revealed that the application of external forces to E-cadherin–based adhesions stimulates liver kinase B1 (LKB1), leading to the recruitment and activation of AMPK at apical junctions. Importantly, activated, junctional AMPK then stimulates glucose uptake, ATP production, Rho activation, and myosin 2 activity, all of which serve to reinforce the stressed junction. Although the precise pathway linking LKB1/AMPK activation to myosin 2 activation has not been fully elucidated, it could involve direct phosphorylation of myosin 2 by AMPK ^29^, inactivation of a key myosin phosphatase ^30^, or activation of Rho guanine nucleotide exchange factors (GEFs) ^31^. In a related study, Saito *et al*. linked AMPK and myosin 2 to glucose uptake in C2C12 myotubes ^32^. They found that the adaptor protein APPL1 induces AMPK-dependent glucose uptake in response to stretch. Interestingly, APPL1 expression also stimulated the association of PKCzeta with myosin 2, and myosin 2 inhibition blocked glucose uptake.

Interplay between AMPK and cell mechanics could also be occurring at sites of cell–matrix interaction and cell protrusion because AMPK is found at focal adhesions ^33^ and is activated at the leading edge of migratory cells ^34^ where integrin activation is robust. Although it remains unclear whether actin-based protrusions or actomyosin-mediated contractions are actually activating AMPK, Cunniff *et al*. suggest that the activation of AMPK at the leading edge of migratory ovarian cancer cells is necessary to stimulate mitochondrial transport into that region so as to increase local ATP production in support of actin dynamics ^34^.

### Mitochondrial homeostasis

In addition to actomyosin-sensitive intermediaries that might influence mitochondrial activity, direct interactions between actomyosin and mitochondria might play a role in mitochondrial quality control. Traditionally, most mitochondria–cytoskeleton interactions have focused on microtubules and microtubule-associated motors as the critical determinants of mitochondria position, transport, and function (reviewed in 35). More recently, actin, myosin motors, and cell mechanics have entered the fold ^36^. In addition to transport and anchoring roles suggested for myosin 5, myosin 6 ^37^, and myosin 19 ^38, 39^, myosin 2 has been implicated in mediating mitochondrial fission, an essential element of mitochondrial quality control ^40^. Specifically, Korobova *et al*. found that inhibiting myosin 2 results in increased mitochondrial length, indicative of a defect in fission, and that myosin 2 localizes to mitochondria fission sites in an actin-dependent manner ^40^. Considering these and other data, they proposed that actin and myosin 2 form a mini-contractile ring around the mitochondria which drives its fission in conjunction with dynamin. How such a small ring could be created is unclear, however, as the length of just one myosin 2 bipolar filament (~300 nm) is roughly the same size as the mitochondrion undergoing constriction. Recent work by Yang and Svitkina sheds light on this issue ^41^. They demonstrated that, whereas light microscopy resolves myosin 2 at constriction sites, electron microscopy reveals ultrastructural detail and that myosin 2 actually localizes adjacent to constriction sites. Considering these and other data, they propose that dynamic and stochastic deformations of the mitochondria driven by myosin 2–dependent contractions occurring in the general vicinity initiate curvature-sensing mechanisms that drive dynamin-dependent constriction ^42– 44^.

### Lipid storage

Two recent studies have implicated actomyosin in lipid synthesis and storage. In the first study, Romani *et al*. performed an unbiased metabolomics screen in breast cancer cell lines plated on soft substrates or after inhibiting myosin 2 ^45^. In both cases, the authors found an accumulation of neutral lipid synthesis relative to untreated cells on stiff substrates. This accumulation was independent of YAP/TAZ, mTOR, and AMPK but dependent on the transcription factor SREBP. Additional experiments led to a model in which extracellular matrix stiffness and actomyosin tension increase the stiffness of the Golgi by an unknown mechanism. In the absence of actomyosin-mediated Golgi stiffening, Arf1 and Lipin-1 can no longer inhibit SCAP/SREBP translocation from the endoplasmic reticulum to the Golgi and subsequent SREBP activation to drive the upregulation of fatty acid and cholesterol synthesis genes. Interestingly, the increase in neutral lipids could also be due to the direct interaction of actomyosin with lipid droplets, as Pfisterer *et al*. have shown that myosin 2 and the formin FMNL1 localize to lipid droplets and are required for the dissociation of adjacent lipid droplets ^46^. Moreover, inhibition of myosin 2 results in enlarged lipid droplets because of the decreased accessibility of lipid-modifying enzymes and subsequent triacylglycerol accumulation.

### Cancer cell metastasis

Two hallmarks of cancer are tissue stiffening and an increase in energy consumption to support enhanced proliferation. Although the mechanisms by which individual cancer cells alter their metabolism in response to changes in tissue stiffness vary greatly, cells with a greater metastatic potential appear to have increased metabolic plasticity ^47, 48^. In other words, if a cancer cell can make use of diverse fuel sources, then its ability to invade and survive is enhanced. In 2016, Morris *et al*. found that metastatic mammary carcinoma cells display a wider array of metabolic shifts when plated in high-density collagen matrices than do non-metastatic cells ^49^. These shifts included decreased oxygen consumption and glucose metabolism coupled with increased glutamine metabolism to drive the tricarboxylic acid cycle and cellular respiration. Mah *et al*. surveyed an array of cancer cells plated on varying collagen densities or with myosin 2 inhibition ^50^. Using an imaging approach to measure the ratio of NAD to NADH as a readout of glycolysis and oxidative phosphorylation, they found that the most invasive cell lines exhibit an increase in oxidative phosphorylation as the collagen density is increased. Importantly, this metabolic shift was dependent on myosin 2 activity, and less-invasive cell types displayed smaller responses. Collectively then, the elevation in myosin 2–dependent metabolic plasticity observed in response to changes in the mechanical environment might provide a mechanism that allows certain cancer cell types to survive in unfamiliar tissues and to enhance their metastatic potential.

Although many of these recent studies have begun exploring the influence of actomyosin on cell metabolism, an important and related question is, how much ATP does the actomyosin cytoskeleton consume in non-muscle cells? The limited data on this vary widely. One study suggested that actin consumes about 50% of the ATP in unstimulated platelets ^51^, whereas a back-of-the-envelope calculation suggested that cell motility is responsible for less than 1% of ATP consumption ^52^. Clearly, this is an important topic that should be revisited with new technologies.

## Conclusions

Mechanosensitive ion channels have been explored from bacteria to plants to vertebrates. Although the recently discovered Piezo channels are currently and rightfully in the spotlight, the ubiquity and importance of these and other mechanosensitive ion channels mean that many questions remain unexplored. Similarly, although the study of metabolism and energy consumption has fascinated biologists for decades, the links to mechanotransduction are not well defined. Recent efforts to connect mechanics and metabolism have demonstrated crosstalk, but definitive mechanisms remain elusive. Additionally, the intersection of all three fields (cell-generated mechanics, metabolism, and mechanosensitive ion channels) is a fascinating and unexplored topic. For example, is there interplay between mechanosensitive ion channels and metabolism that is modulated by the actomyosin cytoskeleton? Collectively, we look forward to reading many more exciting interdisciplinary studies focused on understanding the role of myosin 2 in environmental sensing.
